# The Physical Mechanism for Retinal Discrete Dark Noise: Thermal Activation or Cellular Ultraweak Photon Emission?

**DOI:** 10.1371/journal.pone.0148336

**Published:** 2016-03-07

**Authors:** Vahid Salari, Felix Scholkmann, Istvan Bokkon, Farhad Shahbazi, Jack Tuszynski

**Affiliations:** 1 Department of Physics, Isfahan University of Technology, Isfahan 84156-83111, Iran; 2 School of Physics, Institute for Research in Fundamental Sciences (IPM), Tehran 19395-5531, Iran; 3 Biomedical Optics Research Laboratory, Department of Neonatology, University Hospital Zurich, University of Zurich, 8091 Zurich, Switzerland; 4 Research Office for Complex Physical and Biological Systems (ROCoS), 8038 Zurich, Switzerland; 5 Vision Research Institute, 25 Rita Street, Lowell, MA 01854, United States of America; 6 Psychoszomatic OutPatient Department of the National Center for Spinal Disorders, Budapest H-1126, Hungary; 7 Department of Physics, University of Alberta, T6G 2J1, Edmonton, AB, Canada; Dalhousie University, CANADA

## Abstract

For several decades the physical mechanism underlying discrete dark noise of photoreceptors in the eye has remained highly controversial and poorly understood. It is known that the Arrhenius equation, which is based on the Boltzmann distribution for thermal activation, can model only a part (e.g. half of the activation energy) of the retinal dark noise experimentally observed for vertebrate rod and cone pigments. Using the Hinshelwood distribution instead of the Boltzmann distribution in the Arrhenius equation has been proposed as a solution to the problem. Here, we show that the using the Hinshelwood distribution does not solve the problem completely. As the discrete components of noise are indistinguishable in shape and duration from those produced by real photon induced photo-isomerization, the retinal discrete dark noise is most likely due to ‘internal photons’ inside cells and not due to thermal activation of visual pigments. Indeed, all living cells exhibit spontaneous ultraweak photon emission (UPE), mainly in the optical wavelength range, i.e., 350–700 nm. We show here that the retinal discrete dark noise has a similar rate as UPE and therefore dark noise is most likely due to spontaneous cellular UPE and not due to thermal activation.

## Introduction

Photoreceptor cells have two components of the dark noise: a continuously low amplitude component (≈ 0.2 pA) and a spontaneous discrete component (≈ 1 pA) [1]. For half a century, the mechanism underlying discrete dark noise of photoreceptors in the eye has remained highly controversial and poorly understood [2]. The main question is: Why is there spiking activity of photoreceptors when there is no photon absorbed by it [3]? This spiking reduces the sensitivity of vision and is referred as a false signal [3].

The discrete components of noise are indistinguishable in shape and duration from those produced by real photon induced photoisomerization [2]. Baylor et al. [4] calculated the activation energy of the thermal process in toad rhodopsin to be about 22 kcal/mol. However, this numerical value is in conflict with the significantly less required energy for activation by a single photon, i.e. 40–50 kcal/mol [5]. To resolve this discrepancy, various mechanisms were proposed such as the existence of a subpopulation of molecules with an unprotonated Schiff base in vertebrate rods [6], structural fluctuations in the protein structure [7], and the incorporation of a quantum chemical model [8]. However, to date, most of the models of the discrete components of rod noise are essentially based on thermal processes [2, 8, 9]. The application of the conventional Arrhenius equation

$$k=Af_{B}=Ae^{\frac{-E_{a,B}}{RT}},$$ (1)

where *k* is the rate constant, *A* the pre-exponential factor and *f*_*B*_ represents the Boltzmann distribution ($f_{B}=e^{\frac{-E_{a,B}}{RT}}$, where *E*_*a*,*B*_ is the Boltzmann activation energy, *R* is the gas constant and *T* is the absolute temperature) to model the temperature dependence of dark noise results in the fact that the predicted thermal activation energy amounts to only a part of the photo-isomerization activation energy measured experimentally [4, 5, 9]. This leads to the conclusion that the molecular pathway due to spontaneous thermal activation is different from that due to photo-activation [1].

Recently, the validity of using the Boltzmann distribution in this context has been debated [2, 8]. Consequently, it has been proposed [2] that due to thermal activation of the low energy vibrational modes, the Hinshelwood distribution, i.e. $f_{H}=e^{\frac{-E_{a,H}}{RT}}\sum_{1}^{m}\frac{1}{(m-1)!}{\left(\frac{E_{a,H}}{RT}\right)}^{m-1}$, where *E*_*a*,*H*_ is the Hinshelwood activation energy, *m* is the number of molecular vibrational modes, contributing thermal energy to pigment activation, should be used instead of the Boltzmann distribution and thus the Arrhenius equation should be written in the following form:

$$k=Af_{H}=Ae^{\frac{-E_{a,H}}{RT}}\sum_{1}^{m}\frac{1}{(m-1)!}{\left(\frac{E_{a,H}}{RT}\right)}^{m-1}.$$ (2)

The main aim of the present paper is to show that the thermal activation based on the Hinshelwood distribution proposed primarily by Ala-Laurila et al. [9], and extensively discussed by Luo et al. [2] and supported by Gozem et al. [8], is unlikely to be the main source of the photoreceptor’s dark noise. Specifically, we analyze in detail the calculations presented in the work by Luo et al. [2] (i.e. the most important paper in the literature on this topic) and identify several shortcomings which question the validity of explaining the dark noise of rods and cones by only assuming a thermal activation energy process.

Finally, we demonstrate that the retinal discrete dark noise can be due to spontaneous cellular ultraweak photon emission (UPE) in photoreceptor cells that have the same rates as dark noise rates. Our proposed solution to this problem of discrete dark noise in photoreceptors is based on simple testable assumptions and is mathematically self consistent.

## A critical evaluation of using the Hinshelwood distribution to model the dark noise in photoreceptors

It has to be noted that the application of the Hinshelwood distribution to model one molecule, $f_{H}=e^{\frac{-E_{a,H}}{k_{B}T}}\sum_{1}^{m}\frac{1}{(m-1)!}{\left(\frac{E_{a,H}}{k_{B}T}\right)}^{m-1}$ where *k*_*B*_ is the Boltzmann constant, is only valid in the classical limit where the thermal energy scale is much larger than the energy level spacing (*ϵ*) of the quadratic modes of the molecule (i.e. *k*_*B*_ *T* ≫ *ϵ*). Hence, assuming room temperature at which the thermal energy is about 25 meV, there must exist many modes with much less energies than this value. However, the opposite is true since the resonance Raman excitation of rhodopsin reveals that the Raman lines corresponds to several tens of modes with energies varying from 98–1655 cm^−1^ (corresponding to ∼ 10–200 meV, respectively) which are in order or larger than the scale of the thermal energy [10–12]. Moreover, Luo et al obtained 45 modes that have equal energy values, *k*_*B*_ *T* (“each vibrational mode of the molecule contributing a nominal energy of *k*_*B*_ *T*”) [2] in which the 45 modes all are activated and each energy mode has exactly the same energy as the thermal energy. As a conclusion, the equipartition theorem [13] cannot be applied for these modes; hence the application of the Hinshelwood distribution to model the dark noise of photoreceptors is questionable.

## A reassessment of the number of modes and the pre-exponential factor in the Arrhenius equation

Even if we agree that the Hinshelwood distribution is applicable for photoreceptors then the methodology and the obtained results by Luo et al can be questioned. The rate of change of the term ln *k* with temperature, ∂ ln *k*/∂*T* according to the ‘conventional Arrhenius’ model (i.e. Eq 1) is [9]

$$\frac{\partial \ln k}{\partial T}=\frac{E_{a,B}}{RT^{2}}.$$ (3)

On the other side, the following expression for *k* derives from the last term of Eq 2 in the limit *E* ≫ *RT* [9],

$$k=Af_{H}=A\frac{e^{\frac{-E_{a,H}}{RT}}}{(m-1)!}{\left(\frac{E_{a,H}}{RT}\right)}^{m-1},$$ (4)

which is a good approximation for the full series in Eq 2, and thus the corresponding rate of change according to the ‘Hinshelwood’ model (based on Eq 4) is [9]

$$\frac{\partial lnk}{\partial T}=\frac{E_{a,H}-\left(m-1\right)RT}{RT^{2}},$$ (5)

and consequently Eq 6 is obtained for the difference between the Hinshelwood and Boltzmann activation energies [2, 9, 14]

$$E_{a,H}-E_{a,B}=\left(m-1\right)RT,$$ (6)

where the activation energies are equal only for *m* = 1. The Eq 6 has four parameters in general (*E*_*a*,*H*_, *E*_*a*,*B*_, *m*, *T*) where *m* is the number of molecular vibrational modes and it is obtained based on the other three parameters. In fact, Luo et al. [2] determined *m* based on the Eq 6, only for Bufo red rhodopsin and applied it (i.e., *m* = 45) to all types of photoreceptors. The *m* value for Bufo red rhodopsin with *λ*_*max*_ = 500 nm is obtained based on the above equation where *E*_*a*,*H*_ is the thermal isomerization activation energy of 48.03 kcal/mol, *E*_*a*,*B*_ is the apparent thermal activation energy of 21.9 kcal/mol at absolute temperature *T* = 296 K [2], and *R* is the universal gas constant. If we consider mouse rhodopsin with the same *λ*_*max*_ value as the Bufo rhodopsin, i.e. *λ*_*max*_ = 500 nm, and use the apparent thermal activation energy of 14.54 kcal/mol [2] we find *m* = 58, which is different than the generally valid relation *m* = 45. In addition, a value of *m* different from 45 and 58 is obtained for *A*_1_ human red cones (see Table 1).

**Table 1:** The pigments given in the Luo et al’s work [2] have been revised based on the apparent thermal energy, *E*_*a*,*B*_, and the *m* value. It is seen that the *A*_1_ Bufo rhodopsin and the *A*_1_ mouse rhodopsin have similar *λ*_*max*_ values (*λ*_*max*_ = 500 nm) while their *E*_*a*,*B*_ were different. This causes a significant difference in the *m* values. Another *m* value is obtained for the *A*_1_ human red cone, which is again different than the ‘exclusive’ value *m* = 45. These results indicate that the *m* = 45 value is not a unique value for the all pigments.

Pigment	*λ*_*max*_ (nm)	*E*_*a*,*H*_(kcal mol^−1^)	Measured rate constant (s^−1^)	*E*_*a*,*B*_(kcal mol ^−1^)	*m*
*A*_1_ Bufo rhodopsin	500	48.03	4.18 × 10^−12^	21.9	45
*A*_1_ Mouse rhodopsin	500	48.03	6.64 × 10^−11^	14.54	58
*A*_2_ Xenopus rhodopsin	521	46.10	3.70 × 10^−11^	Not specified	−
*A*_1_ Human red cone	557	43.12	4.14 × 10^−8^	14.64	50
*A*_2_ Human red cone	617	38.93	6.70 × 10^−7^	Not specified	−
*A*_1_ Bufo blue cone	432	55.59	9.39 × 10^−14^	Not specified	−

In the above cases, the apparent thermal energy values were taken from the paper of Luo et al. [2], so their statement regarding the general validity of the parameter value *m* = 45 is not supported by experimental findings. The authors determined that the number of modes (i.e. *m* = 45) is generally valid for the all values of *λ*_*max*_ where the activation energy of the photoreceptor molecule has been experimentally shown by Luo et al. [2] to be $E_{a}^{T}=\frac{0.8hc}{\lambda_{max}}$ where $E_{a}^{T}$ is the activation energy of the pigment, *h* is the Planck’s constant, *c* is the speed of light, and *λ*_*max*_ is the maximum required wavelength of photon to activate the visual pigment. Thus, the Arrhenius equation by assuming $E_{a,H}=E_{a}^{T}$ can be written as follows:

$$k=Ae^{-\frac{0.8hc}{\lambda_{max}RT}}\sum_{1}^{m}\frac{1}{(m-1)!}{\left(\frac{0.8hc}{\lambda_{max}RT}\right)}^{m-1}.$$ (7)

**Fig 1:**
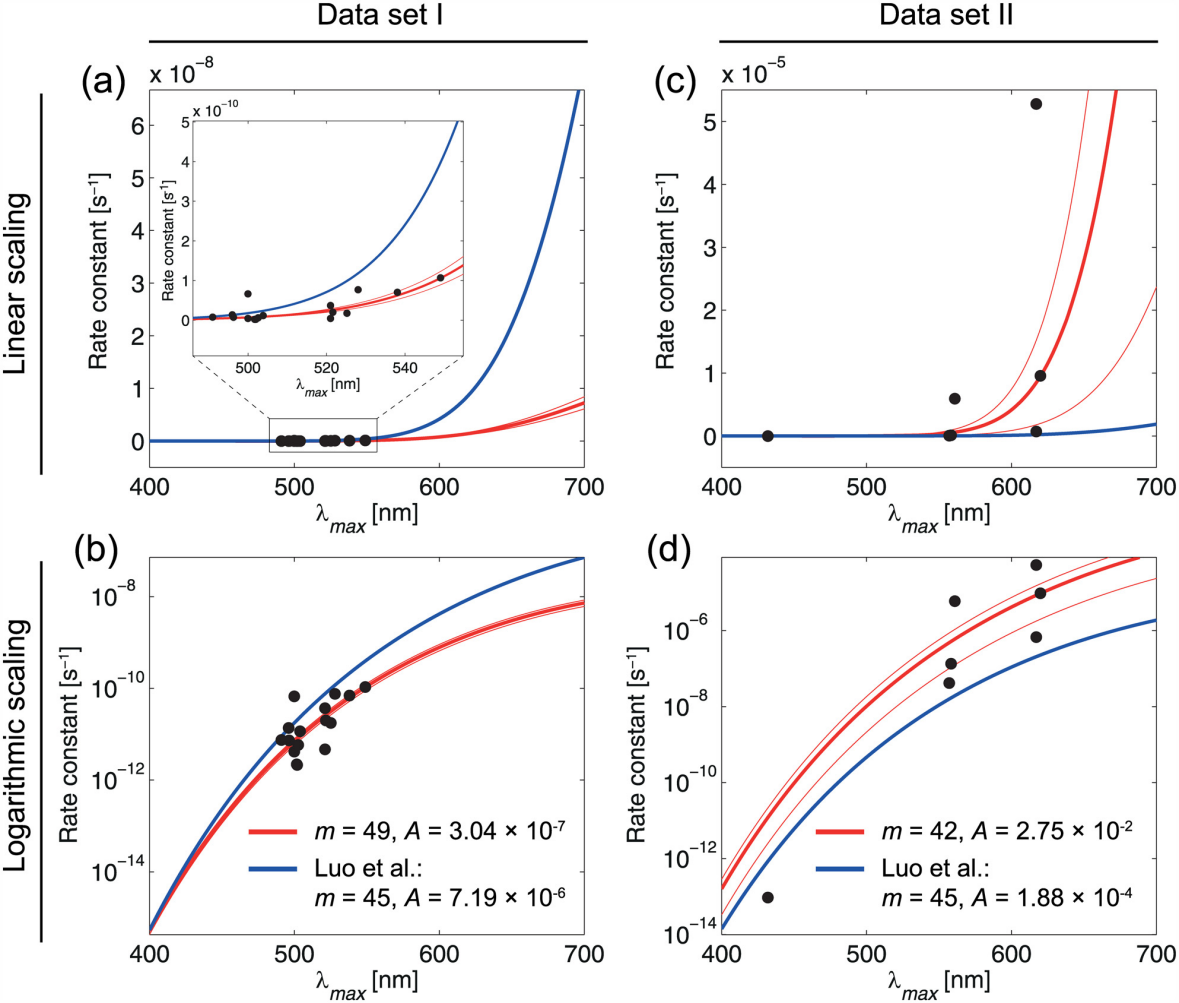
Fitted functions (rate constant *vs* λ_*max*_) according to Eq (7) with optimal *m*-values (red), and *m* = 45 (blue) as predicted by Luo et al [2]. Shown are the results with a linear (a, c) and logarithmic scaling (b, d). It is seen that *m* = 45 is not an optimal value relative to *m* = 49 for rods and *m* = 42 for cones.

To show the discrepancy more clearly, the rate constant diagrams based on Eq 7 for *m* = 45 and for our obtained *m* values, *m* = 49 for rod cells and *m* = 42 for cone cells, based on the fitting method [15] are compared with the experimental data for different rod and cone cells in Fig 1. The results indicate that *m* = 45 is not an exclusive value and has a significant deviation relative to the experimental data. Moreover, our obtained pre-exponential factors (*A*) deviate from the *A* value used by Luo et al which was obtained by the authors by simple averaging and not by fitting, which is imprecise as well.

As a matter of fact, the key argument regarding the trade-off between the number of vibrational modes and the energy barrier has been introduced in the original paper by Ala-Laurila et al. [9] (and not Luo et al. [2]) promoting the use of the Hinshelwood model to understand the correlation between the spectral sensitivity and dark event rates of visual pigments. The criticism presented in this section was also mentioned in the above original paper [9] when developing the model where it clearly makes the following point: “In the model parameters, energy offsets are traded against changes in the apparent number of thermal modes (*n*/2) involved in the activation process. Due to this trade-off, the model is not very helpful for determining the precise relation between *E*_*a*,*H*_ and *E*_*a*_, nor the value of *n* from the experimental data”.

### Determination of the optimal number of modes

The optimal number of molecular vibrational modes (*m*) contributing thermal energy to the pigments activation was found by (i) computing Eq 2 for *m* = 35, 36, …, 65 (data set I, see Table 2) and *m* = 42, 43, …, 49 (data set II, see Table 3), respectively; (ii) fitting the functions with the free parameter A to the data sets; and (iii) determining the goodness of fit by calculating the root-mean-squared error (RMSE).

**Table 2:** Data set I consisting of rods and rhodopsins. Data are taken from Luo et al. [2], which are measured/obtained at 23°C, and from Ala-Laurila et al. [9] at 21°C. It can be simply shown that the difference between measurements at these two temperatures is trivial and does not affect the values.

Species, type	*λ*_*max*_ (nm)	Measured rate constant (s^−1^)
Bufo, rhodopsin	500	4.18 × 10^−12^
Mouse, rhodopsin	500	6.64 × 10^−11^
Xenopus, rhodopsin	521	3.70 × 10^−11^
Salamander, rhodopsin	502	2.13 × 10^−12^
Salamander, rhodopsin	528	7.66 × 10^−11^
Macaque (Macacafascicularis), rods	491	7.45 × 10^−12^
Dogfish (Scyliorhinuscanicula), rods	496	1.36 × 10^−11^
Human, rods	496.3	7.30 × 10^−12^
Bullfrog (Ranacatesbeiana), rhodopsin	501.7	2.21 × 10^−12^
Common toad (Bufobufo), red rods	502.6	5.86 × 10^−12^
Cane toad (Bufomarinus), red rods	503.9	1.17 × 10^−11^
Larval tiger salamander (Ambystomatigrinum)(A2), rods	521	4.69 × 10^−12^
Clawed frog (Xenopuslaevis), rods	521.6	2.00 × 10^−11^
Bullfrog (Ranacatesbeiana) porphyropsin rods	525.2	1.76 × 10^−11^
Hybrid sturgeon (Husohuso X Acipensernudiventris) rods	538	7.00 × 10^−11^
Sturgeon (Acipenserbaeri) rods	549	1.07 × 10^−10^

**Table 3:** Data set II consisting of cone pigments. Data are taken from Luo et al. [2] and Ala-Laurila et al. [9].

Species, type	*λ*_*max*_(nm)	Measured rate constant (s^−1^)
Human, red cone	617	6.70 × 10^−7^
Turtle (Trachemysscriptaelegans), L-cone	617	5.28 × 10^−5^
Human, red cone	557	4.14 × 10^−8^
Bufo, blue cone	432	9.39 × 10^−14^
Salamander, cone	557	4.14 × 10^−8^
Human, L-cone	558.4	1.34 × 10^−7^
Macaque (Macacafascicularis), L-cone	561	5.94 × 10^−6^
Larval tiger salamander (Ambystomatigrinum), L-cone	620	9.58 × 10^−6^

**Fig 2:**
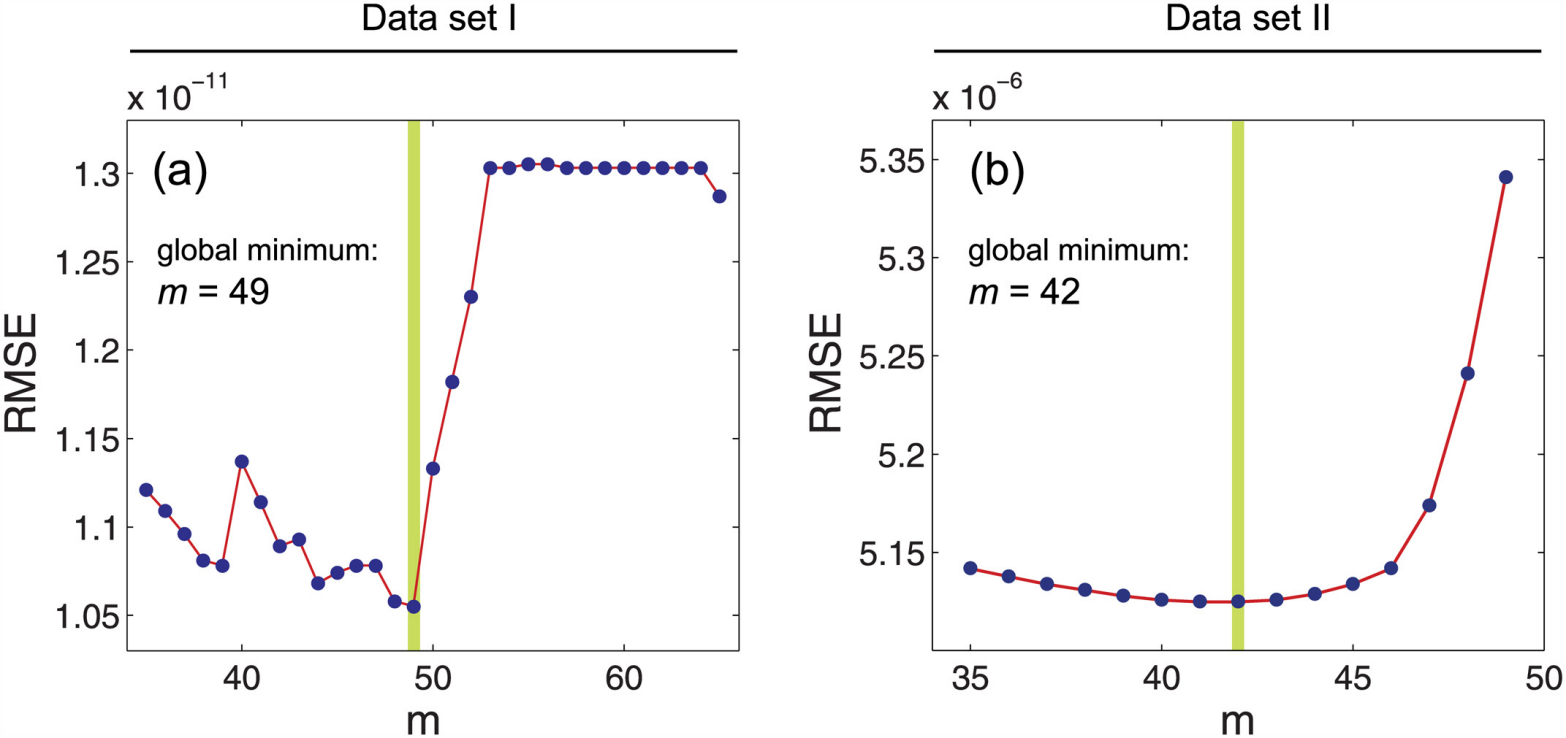
A robust nonlinear least squares fitting with the least absolute residuals (LAR) method and the Levenberg-Marquardt algorithm (LMA) are used to obtain the RMSE values for the curve fitting with the Eq (2) for *T* = 296 K, performed on the rods in the data set I (a), and cones in the data set II (b). The global minima are highlighted as green vertical lines where *m* = 49 is obtained for rods and *m* = 42 is obtained for cones.

**Fig 3:**
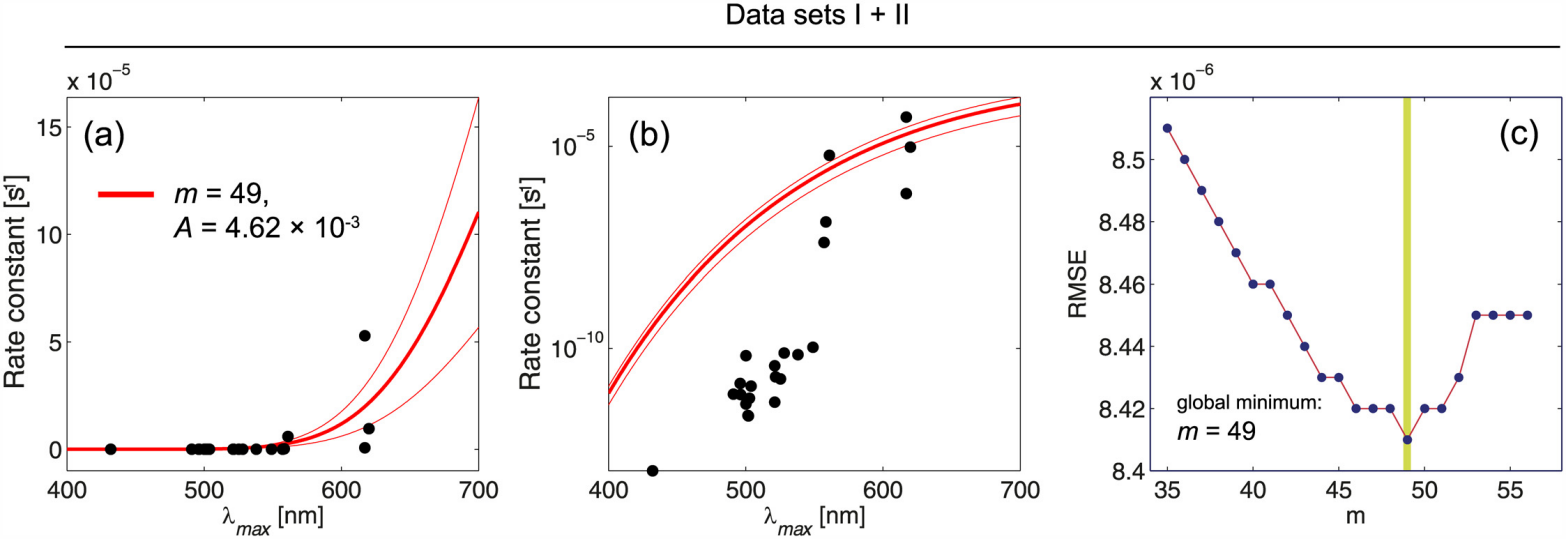
Results of fitting the Eq (7) to the combined data sets (I and II) with linear (a), and logarithmic scaling (b). The RMSE values are obtained for *m* = 35, 36, …, 56 (c). The best *m* and *A* values for the combination of data sets I, II are obtained, i.e. *m* = 49 and *A* = 4.62 × 10^−3^. The combined data points shows a large amount of discrepancy with the fitted Arrhenius equation based on the Hinshelwood distribution, which indicates that there is no a unique Arrhenius equation based on the Hinshelwood distribution with a unique *m* value to satisfy the all experimental data.

The function with the lowest RMSE value was then chosen as the function describing the relationship between *λ*_*max*_ and the rate constant in the best way. A robust nonlinear least squares fitting with the least absolute residuals (LAR) method [16] and the Levenberg-Marquardt algorithm (LMA) [15, 17] was used. The advantage of LAR over ordinary least squares (OLS) is that the method is more robust against deviations from the normality assumption of the data. LMA combines the advantages of gradient-descent and Gauss-Newton methods in order to determine a global minimum of a function. The best fit is obtained for *m* = 49 (data set I, RMSE = 1.055 × 10^−11^, A = 3.04 × 10^−7^) and *m* = 42 (data set II, RMSE = 5.13 × 10^−6^, A = 2.75 × 10^−2^), respectively (see Fig 2). The results obtained by Luo et al. [2], the functions for m = 45 and A = 7.19 × 10^−6^ (rhodopsins) and *A* = 1.88 × 10^−4^ (cone pigments), were plotted, too. Fig 3 shows the fitted functions for the optimal m-values for data sets I and II. In addition to the optimal functions, the function for m = 45 was plotted as well as the 95 percent confidence bound of the fitting procedure. The functions were plotted in Fig 1 with a linear scale (a, b) as well as with a logarithmic one (c, d).

If we apply the average of *m* values, 42 and 49, as *m* = 45 with a single *A* value for a combined datasets I and II for both rod and cone cells then the amount of deviation from experimental data will be very large. To check this high deviation please see the predictions of their theory in the following section. As a result, rod and cone cells should be investigated separately with different *m* values.

## Questionable predictions by thermal activation approach

It is claimed that the ratio of rate constants equals to the ratio of their distribution functions [2], i.e.

$$\frac{k_{1}}{k_{2}}=\frac{f_{\geq E_{a}^{T}}^{1}}{f_{\geq E_{a}^{T}}^{2}}.$$ (8)

Since the pre-exponential factor *A* varies with *m* and it varies for different pigments too, it is unfortunately erroneous to compare the distribution ratios (as predicted rate constant ratios) with the measured rate-constant ratios while the *A* values are not equal even for the same number of modes (i.e. *m* = 45) for cone and rod cells [2]. Moreover, the authors have obtained the pre-exponential factors directly from the measured rate constants themselves which causes an unfair comparison. The authors have mentioned that there is about 26-fold difference between *A* values of rods and cones. To check this claim, we compared these ratios for different samples. The results are shown in Table 4, in which very large discrepancies between theory and experiment can be recognized, indicating that the distribution ratios are not equal to the rate constant ratios.

**Table 4:** Comparison between theoretical predictions offered by Luo et al [2] (i.e. $\frac{f_{\geq E_{a}^{T}}^{1}}{f_{\geq E_{a}^{T}}^{2}}$ for *m* = 45, labeled as “Theory”) and the measurements of rate constants of visual pigments (i.e. $\frac{k_{1}}{k_{2}}$, labeled as “Experiment”). Even for similar *λ*_*max*_ values of rods (Bufo and mouse) and cones (human and turtle), 16 and 83 fold difference appeared respectively. For other comparisons between rods and cones the differences are very large numbers which indicates that predicted and measured rate constants are not comparable.

Pigment	*λ*_*max*_(nm)	$E_{a}^{T}$(kcal/mol)	$f_{\geq E_{a}^{T}}$	Theory	Measured rate constant (s^−1^)	Experiment
Bufo rhodopsin	500	48.03	3.65 × 10^−6^	1	4.18 × 10^−12^	$\frac{1}{16}$
Mouse rhodopsin	500	48.03	3.65 × 10^−6^		6.64 × 10^−11^	
Human red cone	617	38.93	2.44 × 10^−3^	1	6.70 × 10^−7^	$\frac{1}{83}$
Turtle(Trachemysscriptaelegans) L-cone	617	38.93	2.44 × 10^−3^		5.28 × 10^−5^	
Larval tiger salamander(Ambystomatigrinum) rod	521	46.10	1.67 × 10^−5^	$\frac{1}{147}$	4.69 × 10^−12^	$\frac{1}{11261261}$
Turtle(Trachemysscriptaelegans) L-cone	617	38.93	2.44 × 10^−3^		5.28 × 10^−5^	
Sturgeon (Acipenserbaeri) rods	549	43.75	7.45 × 10^−5^	$\frac{1}{2}$	1.07 × 10^−10^	$\frac{1}{55555}$
Macaque (Macacafascicularis) L-cone	561	42.82	1.36 × 10^−4^		5.94 × 10^−6^	
Cane toad (Bufomarinus) red rod	503.9	47.67	4.72 × 10^−6^		1.17 × 10^−11^	
Macaque (Macacafascicularis) L-cone	561	42.82	1.36 × 10^−4^	$\frac{1}{29.4}$	5.94 × 10^−6^	$\frac{1}{526315}$

### A short discussion

The most important parameter in this paper is the rate of discrete dark noise, i.e. *k*. If we use the approach of Luo et al (i.e. thermal activation approach by using the Hinshelwood distribution in the Arrhenius equation) the parameter *m* (i.e. the number of vibrational modes) plays the main role. In general, based on the Luo et al approach there are two main equations: The first equation, i.e. Eq 6, has four parameters (*E*_*a*,*H*_, *E*_*a*,*B*_, *m*, *T*) where $E_{a,H}=E_{a}^{T}=0.8hc/\lambda_{max}$ is the required thermal isomerization activation energy that is ideal and obtained theoretically based on the maximum wavelength absorption of the photoreceptor (i.e. *λ*_*max*_). We have shown that the *m* value cannot be unique for the all wavelengths based on the Eq 6. The second equation is the Arrhenius equation, i.e. Eq 2, which gives *k* based on the pre-exponential factor *A*. Thus, the parameters (*E*_*a*,*H*_, *E*_*a*,*B*_, *m*, *T*, *A*, *k*) are the six parameters in the thermal activation approach in which *m* and *k* are the most important parameters. Our critics are mainly against the two above equations that make discrepancies between the theory and experiment (See Figs 1 and 3 and Table 4). We concluded here that there is no a unique Arrhenius equation based on the Hinshelwood distribution to satisfy the all experimental data for rods and cones. Thus, the retinal discrete dark noise cannot be attributed to thermal activation.

## Ultraweak photon emission in the retina

**Fig 4:**
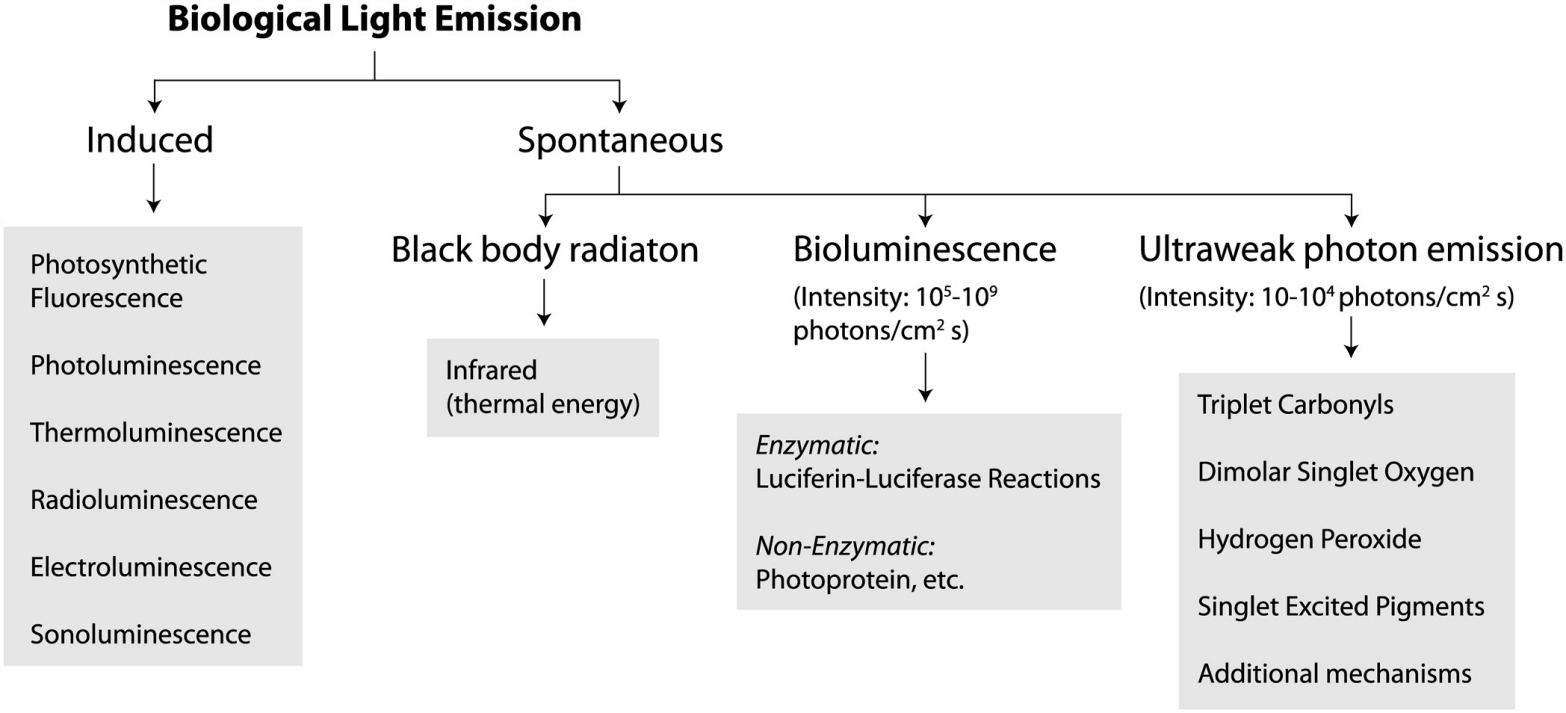
The classification of biological light emission [18–24]. There are two groups of light emissions from biological systems: induced and Spontaneous. In the induced light emission there should be an external excitation such as electric filed, light, heat, ultrasound, etc. But the spontaneous light emission does not need any external excitation and it is classified into three subgroups: Blackbody radiation, Bioluminescence and UPE. The sources in the three subgroups are different. The blackbody radiation at room temperature is in the infrared range and hence it cannot activate visual pigments. The intensity (or rate) of bioluminescence (i.e. 10^5^−10^9^ photons/cm^2^ s) is much higher than the intensity of UPE (i.e. 10–10^4^ photons/cm^2^ s). The rate of retinal discrete dark noise is in the rate range of UPE. UPE is mostly in the visible range that can activate the visual pigments.

Conventional understanding of the human and animal visuals systems holds that the external light signal is transformed into a neural electrical signal by the retina, and then enters into the central nervous system through the optic nerve and produces visual perception. Recent studies have found that UPE may explain some aspects of special visual phenomena [25, 26]. There are two groups of light emissions from biological systems: induced and spontaneous [27, 28]. In the induced light emission there should be an external excitation such as electric filed, light, heat, ultrasound, etc. But the spontaneous light emission does not need any external excitation and it is produced spontaneously due to biochemical reactions in the cells. The spontaneous light is classified into three subgroups: (1) blackbody radiation, (2) bioluminescence and (2) ultraweak photon emission (UPE) [27–29](See Fig 4).

The sources in the three subgroups are different and the intensity (or rate) of bioluminescence (i.e. 10^5^–10^9^ photons/cm^2^ s) is much higher than the intensity of UPE (i.e. 10–10^4^ photons/cm^2^ s). The thermal radiation spontaneously emitted by many ordinary objects can be approximated as blackbody radiation. The radiation has a specific spectrum and intensity that depends only on the temperature of the body. In fact, when the rate of energy absorption is equal to the energy dissipation, the object is said to reach thermal equilibrium with its environment. One path for thermal energy dissipation is through thermal radiation heat transfer. The thermal radiative properties of a blackbody have been studied extensively for many years since 1901 when Max Planck [30] derived the theoretical energy spectrum of blackbody radiation. It has been shown that the intensity of thermal radiation approaches the assumed intensity of UPE (i.e. 1 photon cm^−2^ s^−1^) only in the near infrared spectral region (at 1337 nm for 25°C and at 1280 nm at 37°C) and exceeds it at longer wavelengths [27]. Near room temperature 300 K, a blackbody emitter such as a human body has essentially no power emitted in the visible and near infrared portions of the spectrum but will emit low power radiation at wavelengths predominantly greater than 1*μm*, well outside the visual range of human observation. As a result, the thermal radiation cannot activate the visual pigments.

Bioluminescence is only observed from few special living organisms (e.g. glowworm) but the UPE can be observed from all living cells [27]. The sources in bioluminescence are due to enzymatic (e.g. luciferin-luciferase reactions) and non-enzymatic (e.g. photoprotein) reactions while the sources of UPE is mostly due to reactive oxygen species (ROS) such as triplet carbonyls and singlet oxygen [18, 27]. It has been clearly demonstrated that all living cells (without external excitation) spontaneously and continuously produce UPE [18–24].

The intensity of UPE is on the order of a few, up to 10^4^ photon/(cm^2^ s) (or equivalently 10^−19^ to 10^−14^ W/cm^2^) [18]. UPE is produced from diverse naturally occurring oxidative and biochemical reactions, especially free radical reactions and the simple quenching of excited molecules. The main source of UPE derives from oxidative metabolism of mitochondria and lipid peroxidation that generate photon-emitting molecules such as excited triplet carbonyls R = O* and singlet oxygen ^1^O_2_ [31–33]. Table 5 indicates the spectrum of UPE produced by electronically excited species, which is mostly in the visible region and therefore they can activate visual pigments easily.

**Table 5:** Electronically excited species responsible for UPE.

Electronically excited species	Wavelength
Triplet excited carbonyls	350–550 nm [27]
Singlet excited pigments	360–560 nm [27]
Dimolar singlet oxygen	634 nm, 703 nm [27]
Hydrogen Peroxide	520–650 nm [29]

Recently [34] an experimental evidence has been presented of spontaneous and continuous UPE from freshly isolated rats’ retina. Since the natural lipid peroxidation is one of the main sources of UPE and the photoreceptor cells have the highest oxygen consumption [35] and polyunsaturated fatty acid (PUFA) concentration [36] in the body, there can be a continuous, low level UPE in the retina without any external photonic stimulation. In addition, PUFAs can act directly on the light-sensitive channels and retinal disk membrane phospholipids can be implicated in the control of visual transduction at the molecular level [37]. In reality, UPE exhibits non-linear and Poisson-like distributions [38, 39]. According to Field et al. [40], both additive and multiplicative Poisson noise can make clear behavioral and ganglion cell sensitivity that provides a closer agreement between behavioral and absorptive quantum efficiencies. In the case of vision, sensitivity cannot exceed the limit set by the quantization of light into discrete photons and the consequent Poisson fluctuations in photon absorption. Several aspects of dark-adapted visual processing approach this limit [40].

We have estimated the predicted number of UPE based on the measured retinal discrete dark noise rate given in Table 6, in which a comparison between our estimations and the previously reported UPE from different living systems (i.e. 10^0^ − 10^4^ cm^−2^ s^−1^) [18–24, 27, 31–37, 41, 42] indicates that the dark noise rate is on the order of the UPE rate of photoreceptor cells.

**Table 6:** The experimental measured rates of retinal discrete dark noise in Luo et al’s paper [2] are converted to the unit of (*cm*^−2^ *s*^−1^) to be compared with the rates of UPE. The rates are calculated as *R*_*cm*_ = 10^8^ *R*_*μm*_ in which $R_{\mu m}=\frac{NR_{p}}{S_{\mu m}}$ where *S*_*μm*_ is the area of the measured segment (in terms of *μm*^2^), *S*_*cm*_ is the area of the measured segment (in terms of *cm*^2^), *N* is the number of pigments in the measured segment, *R*_*p*_ is the rate constant per pigment (in terms of *s*^−1^), *R*_*μm*_ is the rate constant in segment (in terms of *μm*^−2^ *s*^−1^), and *R*_*cm*_ is the rate constant in segment (in terms of *cm*^−2^ *s*^−1^). The results indicate that the obtained rates for dark noise are of the order of UPE rate (i.e. a few, up to 10^4^ photons/(*cm*^2^s)). [18–24, 27, 31–37, 41, 42] This results indicate that the retinal discrete dark noise can be potentially due to spontaneous cellular UPE (or biophotons) in photoreceptor cells.

Pigment	*N*	*S*_*μm*_(*μm*^2^)	*R*_*p*_(*s*^−1^)	*R*_*μm*_(*μm*^−2^ *s*^−1^)	*R*_*cm*_(*cm*^−2^ *s*^−1^)(UPE rate?)
*A*_1_ Bufo rhodopsin	6.0 × 10^9^	7.5 × 65	4.18 × 10^−12^	5 × 10^−5^	5 × 10^3^
*A*_2_ Xenopus rhodopsin	2.7 × 10^9^	6.4 × 40	3.70 × 10^−11^	3 × 10^−4^	30 × 10^3^
*A*_1_ Human red cone	6.5 × 10^5^	Not specified [2, 43]	4.14 × 10^−8^	—	—
*A*_2_ Human red cone	8.1 × 10^5^	Not Specified [2, 44]	6.70 × 10^−7^	—	—
*A*_1_ Mouse rhodopsin	6.5 × 10^7^	1.4 × 20	6.64 × 10^−11^	1.5 × 10^−4^	15 × 10^3^
*A*_1_ Bufo blue cone	3.3 × 10^9^	7.3 × 37	9.39 × 10^−14^	14 × 10^−7^	140

To have a comparison between the UPE rate and the dark noise rate we have converted the units of the obtained results by Luo et al to the units of cm^−2^ s^−1^. The rates in the Table 6 are calculated as *R*_*cm*_ = 10^8^ *R*_*μ*_ in which $R_{\mu }=\frac{NR_{p}}{S_{\mu }}$ where *S*_*μ*_ is the area of the measured segment (*μm*^2^), *S*_*cm*_ is the area of the measured segment (*cm*^2^), *N* is the number of pigments in the measured segment, *R*_*p*_ is the rate constant per pigment (s^−1^), *R*_*μ*_ is the rate constant in segment (*μm*^−2^ s^−1^), and *R*_*cm*_ is the rate constant in segment (cm^−2^ s^−1^). In fact, under photopic and scotopic circumstances, the tiny natural UPE from natural retinal lipid peroxidation is negligible, but in dark-adapted retinal cells this evanescent UPE can be measurable. Thus, we conclude that the various examples of discrete dark noise are due to the UPE produced from natural lipid peroxidation in photoreceptor cells.

### UPE depends on temperature

The increased frequency of dark noise in rod cells exposed to higher temperatures is evidence for the thermal contribution to the generation of dark noise [4]. On the other side, UPE directly depends on temperature as well. The retina (photoreceptor outer segments contain rhodopsin) and the brain (neuronal membranes, synapses) have the highest concentration of polyunsaturated fatty acids (PUFA) particularly arachidonic acid (AA, omega-6, 20:4) and docosahexaenoic acid (DHA, omega-3, 22:6) [45, 46]. Since the retinal metabolism is continuously functional, the natural lipid peroxidation also constantly occurs. Mitochondrial respiration chain, lipid peroxidation, free radical reactions are the major sources of UPE production [31, 47, 48]. Since the photoreceptors have one of the highest demands for oxygen, and the photoreceptor outer segments have the highest concentration of PUFA, lipid peroxidation (i.e. temperature dependent process) can be the most important sources of UPE in the retinal system as well as in rod cells. Recently, Kobayashi et al. [49] proved that the the intensity of UPE increased during heating and decreased when the heating was stopped. They also have revealed that the intensity of UPE was dependent on the concentration of reactive oxygens species (ROS), which was not only dependent on the magnitude of stress, but also on the cellular respiration. Temperature is essentially important to all biological functions including synaptic glutamate release. Glutamate, the principal excitatory neurotransmitter in the central nervous system, is distributed widely throughout the neuroaxis. L-glutamate has a significant role in thermoregulation through glutamate receptors [50, 51]. There is a tight association between temperature and glutamate excitotoxicity [52]. It is well known that photoreceptors, bipolar cells and ganglion cells release glutamate [53], which is a temperature dependent process. Experiments by Tang and Dai [22, 34] provided evidence that the glutamate-induced UPE intensity reflects biophoton transmission along the axons and neural circuits.

## Discussion

In this paper we have tried to answer to this question that why there is spiking activity of photoreceptors when there is no external photon absorbed by it? We have considered two possible mechanisms for these false alarms in the eye: (1) thermal energy and (2) spontaneous UPE (or biophotons). In the first case, the Arrhenius equation based on the Boltzmann distribution gives the activation energies of discrete dark noise at a level which is around half the energy for activation in vertebrate rod and cone pigments. Thus, there is a serious inconsistency between the apparent energy barriers of thermal events compared with those found in the photon-driven process. Recently, Luo et al. [2] claimed that they have solved this problem by using the Hinshelwood distribution instead the Boltzmann distribution in the Arrhenius equation to give the correct amount of activation energy. Their approach was also supported by Gozem et al. [8] by proposing a molecular mechanism for thermal activation. In this paper, we have shown that a careful reanalysis of the methodology and results based on the Hinshelwood distribution puts these claims in doubt. We briefly explain the main problems toward the thermal activation approach as follow:

- The Hinshelwood distribution, *f*_*H*_, is not applicable for many-modes activation of visual pigments by heat because the equipartition theorem does not satisfy here.
- The Eq 6 does not give a unique *m* value for the all wavelengths in photoreceptors (See Table 1 and Figs 1 and 3).
- The rate constants ratio is not equal to the distributions ratio (i.e. $\frac{k_{1}}{k_{2}}\neq \frac{f_{\geq E_{a}^{T}}^{1}}{f_{\geq E_{a}^{T}}^{2}}$) because the equality causes a large discrepancy between the predictions in the thermal activation approach (see Table 4).
- There is no a unique Arrhenius equation based on the Hinshelwood distribution to satisfy the all experimental data for rods and cones (See Figs 2 and 3).

On the other hand, the discrete components of noise are indistinguishable in shape and duration from those produced by real photon induced photo-isomerization, so the retinal discrete dark noise is most likely due to ‘photons’ inside cells instead ‘heat’ for thermal activation of visual pigments. There are only three types of spontaneous photon emission from living cells: (1) blackbody radiation, (2) bioluminescence, and (3) UPE. The blackbody radiation is in the infrared range at room temperature and hence it cannot activate visual pigments. The sources in the three mentioned types are different and the intensity of bioluminescence (i.e. 10^5^−10^9^ photons/cm^2^ s) is much higher than the intensity of UPE (i.e. 10–10^4^ photons/cm^2^ s). Bioluminescence is only observed from few special living organisms (e.g. glowworm) but the UPE can be observed from all living cells. It is well known that all living cells (without any excitation) spontaneously and continuously produce UPE. Wang et al. [34] presented the experimental in vitro evidence about the existence of spontaneous UPE from freshly isolated rat’s retina. There is a continuous, low level UPE in the retina without any external photonic stimulation [1].

In fact, the approach of UPE needs only one parameter that is *k*, which makes this solution based on simple testable assumptions and is mathematically self consistent. We have shown that the rates of the discrete dark noise, *k*, are in the range of UPE rates. For example, in the approach of Luo et al the dark noise rates are in terms of ‘counts per photoreceptor per second’, while the previous reported UPE rates are in units of counts/(cm^2^ s) [18–24, 27, 31–37, 41, 42]. We have converted the units of *k* to the units of counts/(cm^2^ s), based on the experimental data of the supplementary materials of the Luo et al paper [2], and have shown that the dark noise rates are of the same order as UPE rates (see Table 6). The spectrum of UPE is mostly in the visible range (see Table 5) that can easily activate visual pigments.

Ala-Laurila et al., [54] replaced the 11-cis-3,4-dehydroretinal (A2) chromophore in salamander rods with the 11-cis-retinal (A1) chromophore that caused an approximately 36-fold decrease in the dark event rate. In fact, the drop in the discrete noise component indicates that the A1 chromophore and the opsin form a pigment that is less susceptible to thermal isomerization than that formed by the A2 chromophore with the same opsin. In all pigments of the Rh1 pigment family, the wavelength of maximum absorption is shorter when the opsin is bound to the A1 chromophore rather than the A2 form [55]. First, visible and NIR spectrum biophotons are linked to electron-excited states. Living cells can produce longer wavelength biophotons easier than shorter wavelength biophotons, since production of shorter wavelength biophotons needs more energy. Second, A1 chromophore and the opsin form a pigment that is less susceptible to thermal isomerization than that formed by the A2 chromophore. This fact can be also relevant for the proposed UPE-induced dark noise, i.e. UPE is able to activate the A1 chromophore structure to a lower degree than the A2 chromophore structure. The different spectral sensitivities of A1 and A2 (max: 502 nm for A1, max: 528 for A2) may play a role, too.

The higher rates of dark noise in cones relative to rods can be explained by the UPE approach. In fact, mitochondria are densely observed in the inner segments of photoreceptor cells. Cones contain more mitochondria than rods. For example, in macaque, mitochondria comprise 74–85% of cone ellipsoids and 54–66% of rod ellipsoids. Since one of the major sources of UPE is derived from mitochondrial oxidative phosphorylation metabolism, this explains why cones produce higher UPE relative to rods [56].

Our arguments for the links between the UPE and the retinal dark noise are briefly as follow:

- The discrete components of noise are indistinguishable in shape and duration from those produced by real photons.
- UPE is mostly in the visible range that can easily activate visual pigments (See Table 5).
- The dark noise has similar rates in the range of UPE in all living cells (See Table 6).
- UPE has been experimentally detected in full darkness from mouse retina.
- UPE is directly temperature dependent (the same as dark noise) which can explain why dark noise rate increases with temperature.
- UPE is a spontaneous process and doesn’t need external excitation (the same as dark noise).
- The UPE approach can explain why cone cells have higher dark noise rates than rod cells.
- The UPE solution is simpler than the thermal energy solution and it is mathematically self-consistent.

Based on the above arguments, we conclude that the retinal discrete dark noise should be attributed to spontaneous cellular UPE but not to thermal noise. In this paper, we have estimated the rates of experimental dark noise based on the units of UPE rates, which confirms the similarity between the UPE rates and the retinal dark noise rates, but the precise number of UPE from each photoreceptor cell (at different temperatures) and its direct link to discrete dark noise is still in need of experimental support. In reality, obtaining the precise number of UPE from photoreceptor cells requires very sensitive and advanced single-photon-detectors with a high signal-to-noise ratio. This will be the focus of our next potential research project and we would like to motivate other research teams to evaluate our approach experimentally.
